# Supplementary material for: A New Approach to the Structure-Properties Relationships Determination for Porous Filled Reinforced Materials
Source: Polymers (Basel). 2022 Oct 18;14(20):4390. doi: 10.3390/polym14204390 (PMC9608482; doi:10.3390/polym14204390)
Supplement: Supplementary file 1 [file polymers-14-04390-s001.zip › polymers-1922019-SM.pdf]

Table S1. List of abbreviations, resp. symbols nomenclature.in this work

Properties	
z_c, z_m	– property without specification for composite material and non-porous matrix
E, E_m, E_c, E_r, E_{cr}	– tensile modulus – generally or in equations for 1-component porous material, for non-porous matrix, for porous filled composite (= composite matrix), for monofilaments (reinforcement) and for porous filled and reinforced composites
σ, σ_m	– strength for porous and non-porous 1-component material
$\sigma_{m,Fmax}$, $\sigma_{c,Fmax}$, $\sigma_{r,Fmax}$, $\sigma_{cr,Fmax}$	– ultimate strength – for non-porous matrix, for porous filled composite (= composite matrix), for monofilaments (reinforcement) and for porous filled and reinforced composites
$\varepsilon_{m,Fmax}$, $\varepsilon_{c,Fmax}$, $\varepsilon_{r,Fmax}$, $\varepsilon_{cr,Fmax}$	– ultimate strain – for non-porous matrix, for porous filled composite (= composite matrix), for monofilaments (reinforcement) and for porous filled and reinforced composites
$A_{m,Fmax}$, $A_{c,Fmax}$, $A_{r,Fmax}$, $A_{cr,Fmax}$	– energy need for ultimate strength achievement – for non-porous matrix, for porous filled composite (= composite matrix), for monofilaments (reinforcement) and for porous filled and reinforced composites
$S_{m,rel}$	– relative area lying below tensile curve up to ultimate strength achievement in case non-porous matrix (obtained by calculation, measured is not available)
Structural parameters	
n, n_c	– porosity, critical porosity
ρ, ρ_c	– density, critical density
v_m, $v_{m(t)}$	– matrix volume fraction – real and with porosity neglecting
n_p	– interspace filling
v_f, $1\text{-}v_f$	– volume fractions of filler, interspace volume
Remaining parameters	
a,p, J, b – up to Eq. (9)	– different parameters used in reference works
b – eq. (12) and further, c	– n_p and $1\text{-}v_f$ parameters exponents ensuring the best fitting of cubic exponential function serving for filled porous composites mechanical behavior description (different properties = different b, c values)
d, e, f, g	– parameters arising through b and c fitting by matrix properties
δ	– OH/NCO rate in polyurethane matrix before curing substituting the matrix polarity and adhesion of matrix to other material components

η – eq. (18)	– reinforcement efficiency in classical mixing rule
η, η_E, η_σ, η_ε, η_A	– correction parameter in our version of mixing rule valid generally, for elastic modulus, ultimate strength, ultimate strain and energy need for ultimate strength achievement
h, h_E, h_σ, h_ε, h_A	– slope of dependence fitting correction parameter η valid generally, for elastic modulus, ultimate strength, ultimate strain and energy need for ultimate strength achievement
k, l	– parameters serving to fitting of h by matrix properties
i, j	– n_p and $1\text{-}v_f$ parameters exponents ensuring the best fitting of cubic exponential function serving for correction parameter η description (different properties = different i, j values)
m, n, o, p	– the same relationship to i and j parameters as d, e, f, g to b and c parameters

Table S2. Meaning of members and mean of their getting for totally all equations occurring in the process of tensile modulus calculation from the calculation of E for porous filled composite serving as matrix to the end (description of parts in relationship describing correction parameter η). This overview should serve for better understanding of the whole calculation process. The other properties use the same calculation method and therefore are not mentioned here.

Parameter	Significance in eq. (13)	Derived from…
E_c	E value of porous filled composite	calculated by Eq. (13)
E_m	E value for non-porous matrix	obtained by fitting in [32]
n_p	interspace filling	Calculated by Eq. (10)
$1\text{-}v_f$	interspace volume	calculated by Eq. (11)
d, e	b exponent parameters	from [32]
f, g	c exponent parameters	from [32]
δ	OH/NCO ratio in matrix before curing	known (adjusted), also from [32]
**Parameter**	**Significance in eq. (19), (20)**	**Derived from…**
E_{cr}	E value for filled reinforced composite	measured
E_c	E value of filled composite	calculated by Eq. (13)
E_r	E value of reinforcement	measured
v_r	volume fraction of reinforcement if porosity is neglected	rate between volumes of reinforcement and whole sample[a)]
η_E	correction parameter	calculated by Eq. (19) or Eq. (20)
**Parameter**	**Significance in eq. (21), (25)–(27)**	**Derived from…**
η_E (21)	correction parameter	calculated by Eq. (19) or Eq. (20)
h_E (21)(25)	E value for non-porous matrix	obtained by fitting in Eq. (21) and then used in Eq. (25)
n_p (21)	interspace filling	calculated by Eq. (10)
$1\text{-}v_f$ (21)	interspace volume	calculated by Eq. (11)
i, j (21), (26),(27)	exponents	values ensuring the highest R^2 in Eq. (21) and then used in Eq. (26) or Eq. (27)

k, l (25)	h_E parameters	fitting of h_E according to E_m in Eq. (25)
m, n (26)	i exponent parameters	*i* fitting according to E_m or h_E in Eq. (26)
o, p (27)	j exponent parameters	*j* fitting according to $E_m \cdot \delta$ or $h_E \cdot \delta$ in Eq. (27)

a) when the porosity is neglected. Calculation is possible due to knowledge of density of components and then of the whole sample.

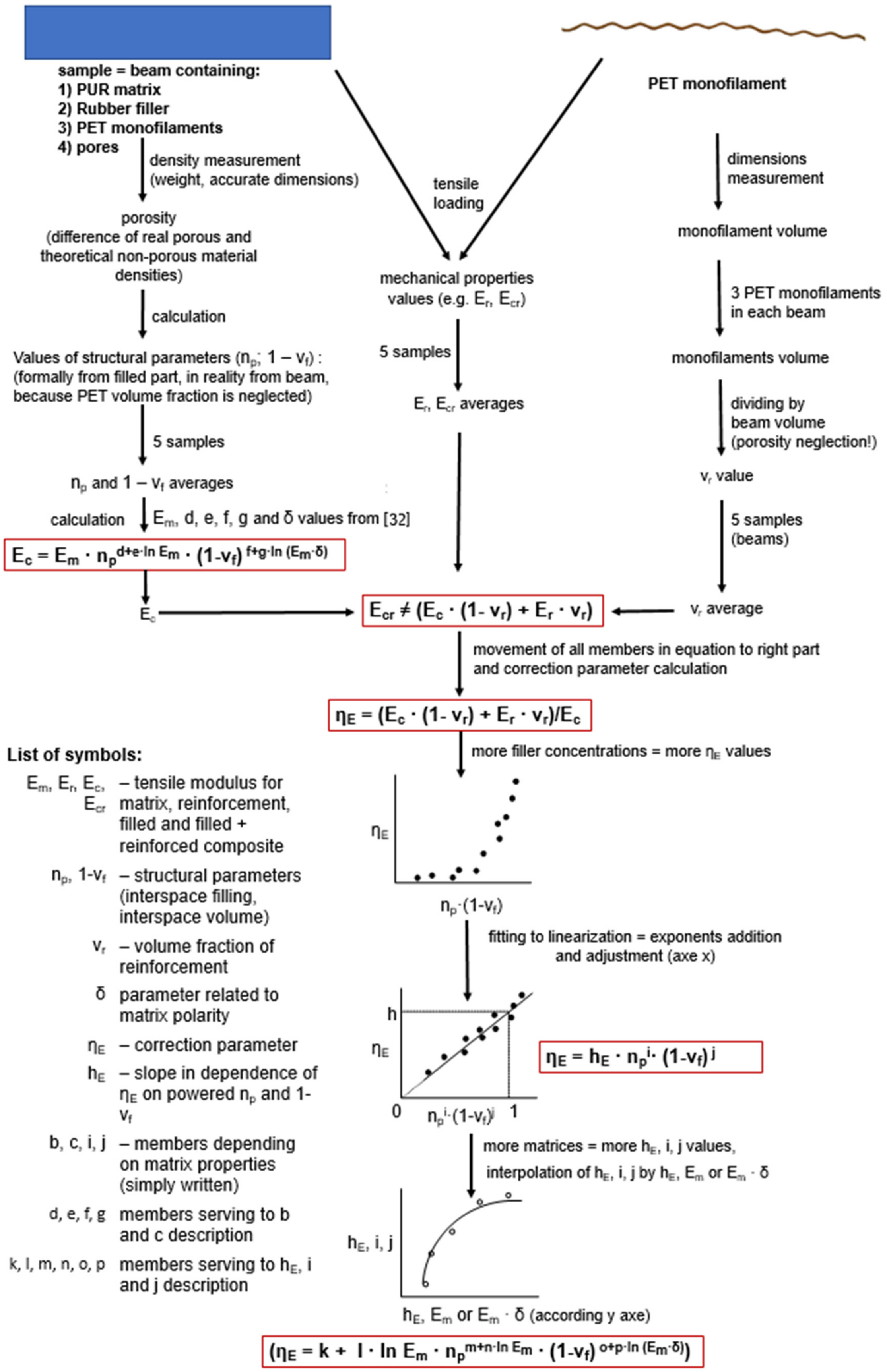

Figure S1. Diagram describing the data processing leading to correction parameter η and description of the correction parameter meaning (further data processing) on the example of tensile modulus.

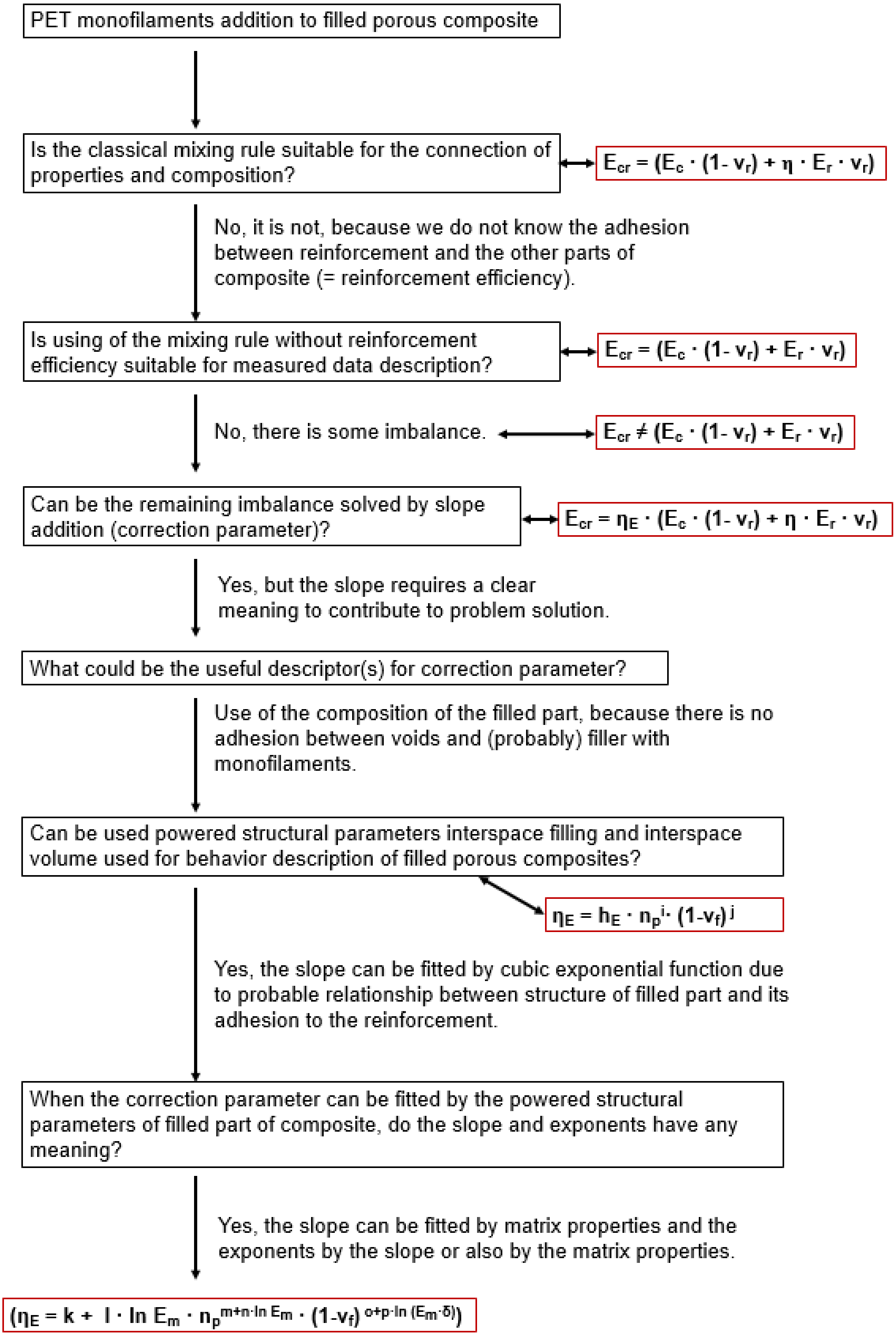

Figure S2. Flow chart describing the process leading to final results – relationships.
